# Supplementary figures and images for: MX2 Viral Substrate Breadth and Inhibitory Activity Are Regulated by Protein Phosphorylation
Source: mBio. 2022 Jul 26;13(4):e01714-22. doi: 10.1128/mbio.01714-22 (PMC9426416; doi:10.1128/mbio.01714-22)

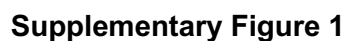

### Supplementary Figure 1

Supplement: FIG S1 [file mbio.01714-22-s0001.pdf]

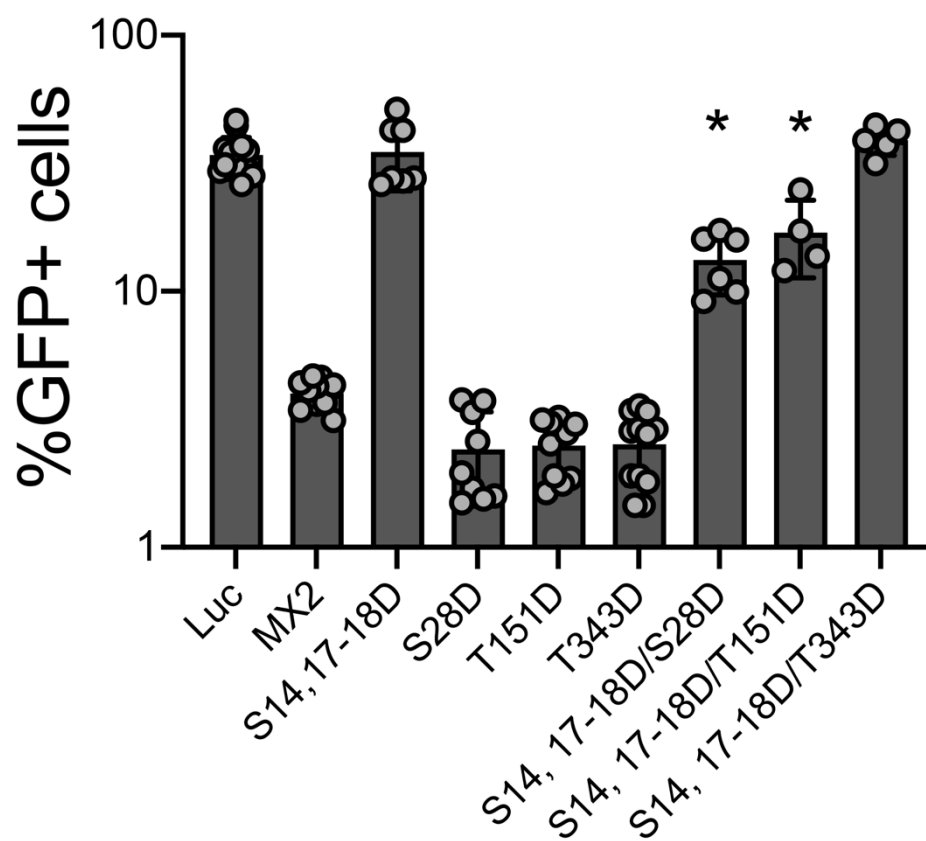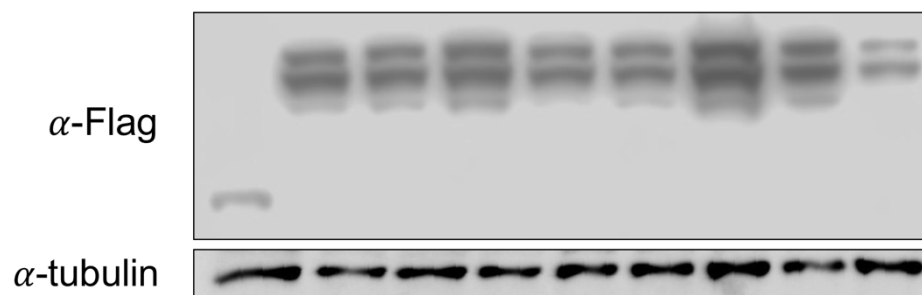

Supplementary Figure 2

Supplement: FIG S2 [file mbio.01714-22-s0002.pdf]

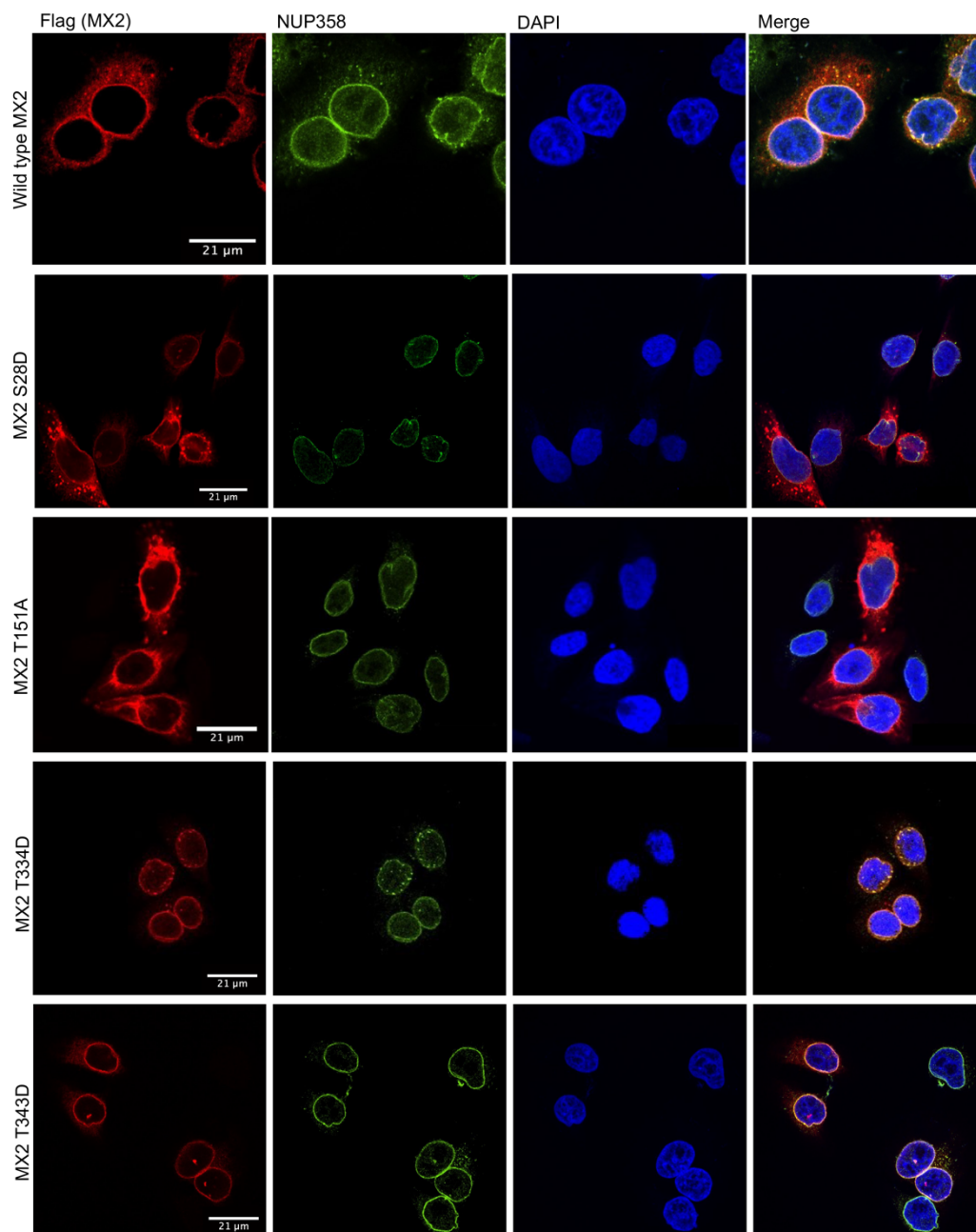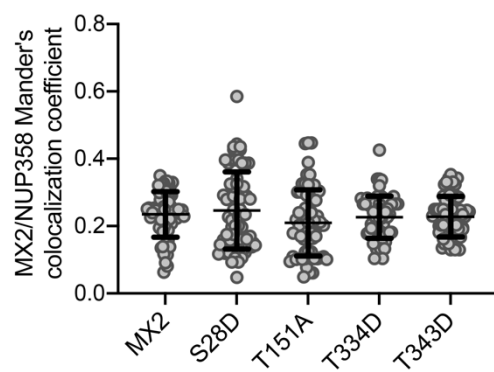

### Supplementary Figure 3

Supplement: FIG S3 [file mbio.01714-22-s0003.pdf]
